# Pectoral Nerve Block II for Cardiac Implantable Electronic Devices

**DOI:** 10.1111/anec.70005

**Published:** 2024-08-15

**Authors:** Shehzad Zafar, Rubaiqa Khan, Muhammad Ali Akbar, Rabia Zameer, Jahanzeb Malik, Waheed Akhtar, Amin Mehmoodi, Muhammad Awais

**Affiliations:** ^1^ Department of Electrophysiology Armed Forces Institute of Cardiology Rawalpindi Pakistan; ^2^ Department of Neurosurgery Rural Health Center Sherwan Abbottabad Pakistan; ^3^ Department of Medicine Sahiwal Teaching Hospital Sahiwal Pakistan; ^4^ Department of Medicine Fatima Jinnah Medical University Lahore Pakistan; ^5^ Department of Cardiology Abbas Institute of Medical Sciences Muzaffrabad Pakistan; ^6^ Department of Medicine Ibn e Seena Hospital Kabul Afghanistan

**Keywords:** cardiac implantable electronic devices, intraoperative local anesthesia, PECS II block, postoperative analgesia, ultrasound‐guided block

## Abstract

**Aim:**

This study aimed to assess the feasibility and effectiveness of the pectoral nerves (PECS) II block in facilitating cardiac implantable electronic device (CIED) insertion in a sample of 120 patients, with a focus on the percentage of cases completed without additional intraoperative local anesthesia.

**Methods:**

PECS II blocks were performed on the left side using ultrasound guidance in all 120 patients. Feasibility was assessed by the proportion of cases completed without the need for extra intraoperative local anesthetic. Secondary outcomes included the amount of additional local anesthetic used, intraoperative opioid requirements, postoperative pain scores, time to first postoperative analgesia, analgesic consumption, patient satisfaction, and block‐related complications.

**Results:**

Of the 120 patients, 78 (65%) required additional intraoperative local anesthetic, with a median volume of 8.2 mL (range 3–13 mL). Fifteen patients (12.5%) needed intraoperative opioid supplementation. Nine patients (7.5%) required postoperative tramadol for pain relief. In total, 98 patients (81.7%) reported high satisfaction levels with the procedure.

**Conclusions:**

The PECS II block, when combined with supplementary local anesthetic, provided effective postoperative analgesia for at least 24 h in 120 patients undergoing CIED insertion. While it did not completely replace surgical anesthesia in most cases, the PECS II block significantly contributed to a smoother intraoperative experience for patients.

## Graphical abstract

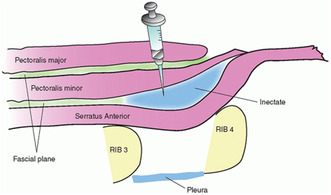

**Abbreviations**
CIED: cardiac implantable electronic device
ICDs: implantable cardioverter defibrillators
IQR: interquartile range
PECS: pectoral nerves
VAS: visual analog scale

## 1 Introduction

Cardiac implantable electronic devices (CIEDs), such as pacemakers, implantable cardioverter defibrillators (ICDs), and cardiac resynchronization therapy devices, play a crucial role in treating various heart conditions, including bradyarrhythmias, ventricular tachyarrhythmias, and advanced systolic heart failure (Harding 2015). With advancements in technology and expanding indications, more patients are receiving these devices (Malik et al. 2021). The placement of CIEDs involves minor surgery, often under local anesthesia, sedation, general anesthesia, or regional anesthesia.

When CIED insertion is performed under local anesthesia, there can be challenges related to inadequate pain relief and patient movement during the procedure (Haas, Richter, and Kubitz 2009). Given that many CIED patients are at high risk for general anesthesia, it becomes important to avoid potential adverse effects associated with it. Some studies and case reports have suggested that certain truncal nerve blocks could offer effective surgical anesthesia and postoperative pain relief for CIED insertion (Mavarez, Ripat, and Suarez 2019; Kilin et al. 2023).

One such technique is the pectoral nerve (PECS) block, consisting of PECS I and PECS II blocks (Moon et al. 2017). These blocks were originally developed for postoperative pain management after breast surgery. The PECS I block targets the lateral surface of the breast and chest wall by injecting local anesthetic between the fascia of the pectoralis major and minor muscles (Blanco, Fajardo, and Parras 2012). The PECS II block is a modification of this technique and can provide analgesia to the axilla region as well, achieved by injecting local anesthetic between the pectoralis minor and serratus anterior muscles. This also blocks the long thoracic and thoracodorsal nerves in addition to the lateral branches of the intercostal nerves (Hoerner et al. 2023).

Interestingly, PECS blocks have shown promise not only in breast surgery but also in cardiac, thoracic surgery, and venous catheter port implantation as part of a multimodal analgesia approach (Ardon et al. 2022; Yalamuri et al. 2017; Renzini et al. 2020). Some studies have even demonstrated the potential use of PECS blocks, either alone or in combination with other nerve blocks, to provide intraoperative analgesia and anesthesia for CIED insertion (Turkmen and Mutlu 2022; Jin et al. 2020).

In summary, the aim of this study was to assess the viability and effectiveness of utilizing the PECS II block for CIED insertion. This technique, originally designed for breast surgery, shows promise in addressing the unique challenges of anesthesia and pain management in CIED patients, particularly those who may not be suitable candidates for general anesthesia. Further research and evaluation are needed to confirm its suitability and benefits in this specific medical context.

## 2 Methods

In this prospective feasibility study, approval was obtained from the Institutional Ethics Committee of the Abbas Institute of Medical Sciences (Study ID # AIMS/23/53), following the principles outlined in the Helsinki Declaration. Patients undergoing CIED insertion, such as pacemakers or ICDs, were recruited for the study, provided they were over 18 years old and met certain criteria. All patients gave written informed consent.

Patients who could not communicate, did not provide written informed consent, had contraindications for the nerve block (like infection at the injection site or coagulation disorders), were hypersensitive to local anesthetics, had a history of surgery or chronic pain at the block site, had a history of alcohol or drug abuse, or were undergoing treatment for psychiatric disorders were excluded from the study.

The primary goal of the study was to determine the feasibility of performing the PECS II block for CIED insertion without the need for additional local anesthesia during surgery. Secondary outcomes included assessing the amount of additional local anesthetic required during the surgery, intraoperative opioid usage, postoperative pain levels, time taken to perform the block, patient and operating physician satisfaction, and any complications related to the block procedure.

The PECS II blocks were administered by an experienced neurosurgeon experienced in this type of analgesia (RK) using an ultrasound‐guided single‐injection technique on the left side (Chakraborty et al. 2016). This involved using a 20‐gauge, 100 mm block needle, and a high‐frequency linear ultrasound transducer. The patient was positioned supine with the arm abducted. The axillary artery and vein were visualized with ultrasound, and the relevant muscles and interfascial planes (pectoralis major, pectoralis minor, and serratus anterior) were identified at the level of the third rib. Utilizing ultrasound, the major landmarks visualized are the pectoralis major muscle, pectoralis minor muscle, and the thoracoacromial artery (pectoral branch). The neurosurgeon then inserted the needle between the serratus anterior and pectoralis minor muscles, injecting 10 mL of 0.5% bupivacaine. Subsequently, the needle was repositioned between the pectoralis major and minor muscles, and an additional 10 mL of 0.5% bupivacaine was injected into the fascial plane.

Block success was confirmed by the patient's complete loss of cold sensation between the T2 and T4 dermatomes. If the patient experienced pain during the procedure, the operating physician could administer 10 mL of additional local anesthetic (lignocaine). In persistent pain, a supplementary IV bolus of 50 mcg fentanyl was given.

All CIED implantations were performed by the same experienced cardiologist, involving a small incision in the left infraclavicular area to create a pocket for device placement and lead insertion.

After surgery, patients were closely monitored for vital signs, including blood pressure and heart rate in the intensive cardiac care unit. Postoperative pain was assessed hourly for the first 24 h using a visual analog scale (VAS). If the VAS score exceeded 3/10, IV tramadol 50 mg was administered. Patient satisfaction was evaluated using a 5‐point Likert scale postoperatively, ranging from “not satisfied” to “very satisfied.”

## 3 Statistical Analysis

In this pilot study, no formal power analysis was conducted due to its preliminary nature. Instead, the researchers relied on insights from previous literature to estimate an appropriate sample size. It was determined, based on this literature, that a sample size ranging from 100 to 140 patients would likely be sufficient to achieve meaningful results (Chakraborty et al. 2016). Consequently, they empirically set an initial goal of enrolling 120 patients in the study.

For the statistical analysis, the researchers utilized SPSS version 26, a commonly used statistical software package. Continuous variables were presented in two ways: either as the mean value plus or minus the standard deviation or as the median value along with the interquartile range (IQR) and range. Categorical data were expressed in terms of absolute frequencies and percentages.

To compare different variables between the two groups, the Student's *t*‐test was applied to those variables that followed a normal distribution, while the Mann–Whitney *U*‐test was used for variables that did not exhibit a normal distribution. The chi‐square test, on the other hand, was employed to analyze categorical data.

To explore potential factors associated with the use of additional local anesthesia during surgical procedures, the researchers conducted a univariate logistic regression analysis. This analysis aimed to identify any variables that might be linked to the need for intraoperative supplemental local anesthetic. A *p*‐value less than 0.05 was considered statistically significant, suggesting a meaningful relationship between the variable under investigation and the use of additional anesthesia. In essence, this approach allowed the researchers to examine and quantify potential factors contributing to the study's outcomes.

## 4 Results

**FIGURE 1:**
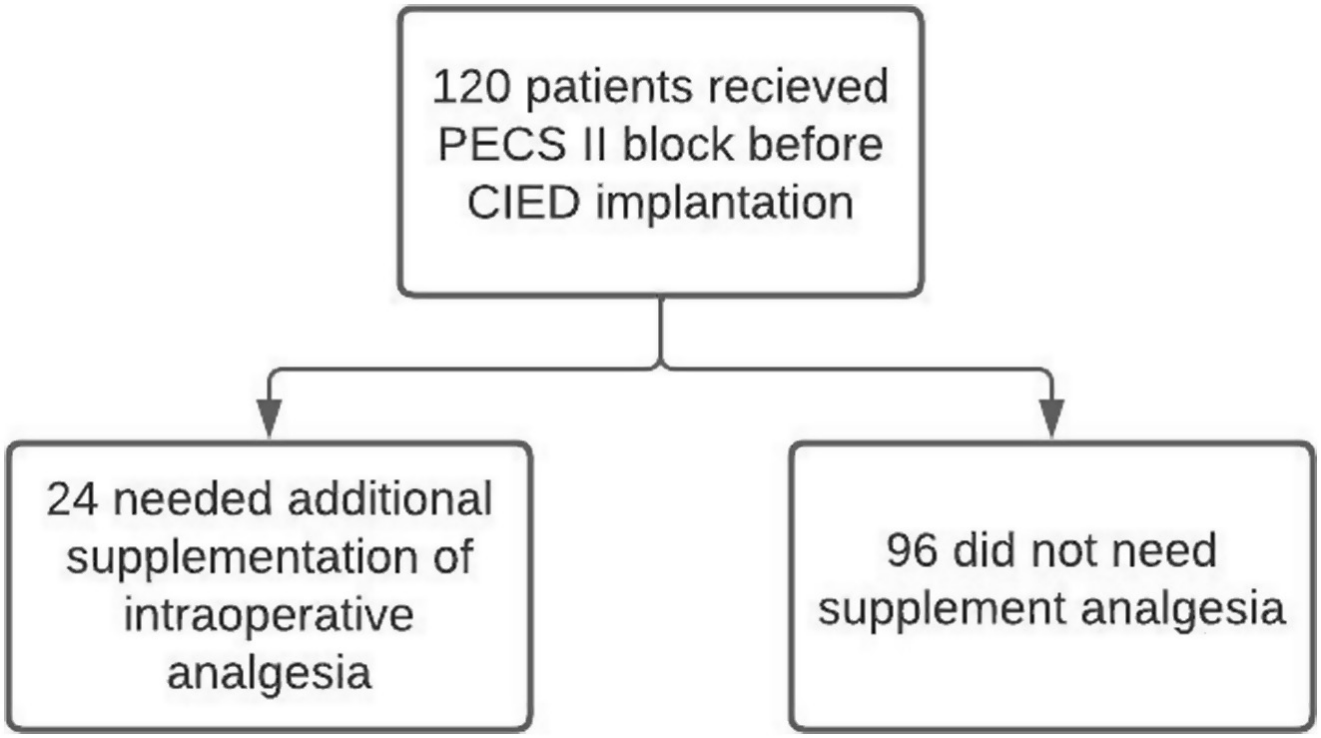
Workflow diagram.

In this cohort of 120 patients, the mean age was 63.2 years (±12.8), with an average BMI of 26.1 kg/m^2^ (±4.2) (Table 1). The majority of patients were male (66.7%) and had an ASA physical status of ASA III (90%). The perioperative data for the 120 patients revealed that the volume of supplemental local anesthetic ranged from 3 to 13 mL, with a median of 8.2 mL (IQR: 5–11.5). A total of 78 patients (65%) required supplemental local anesthetic, 15 (12.5%) required intraoperative fentanyl, and 9 (7.5%) needed tramadol postoperatively (Table 2). The block performance time had a median of 7.8 min (IQR: 6–8.5), and the duration of surgery ranged from 27 to 55 min, with a median of 38 min (IQR: 32–42). Hospital stays were relatively short, with a median of 1.5 days (IQR: 1–2). Figure 1 illustrates a workflow diagram.

**TABLE 1:** Patient characteristics.

Variable (*n* = 120)	Values
Age (years)	63.2 ± 12.8
BMI (kg/m^2^)	26.1 ± 4.2
Gender	
Male	80 (66.7%)
Female	40 (33.3%)
ASA physical status	
ASA II	12 (10%)
ASA III	108 (90%)
EF, %	50.4 ± 8.7
Hypertension	60 (50%)
Coronary artery disease	40 (33.3%)
Diabetes mellitus	30 (25%)
Atrial fibrillation	20 (16.7%)
Chronic renal failure	6 (5%)
Pacemaker	
Single chamber	30 (25%)
Dual chamber	40 (33.3%)
ICD	
Single chamber	50 (41.7%)
Dual chamber	10 (8.3%)

**TABLE 2:** Perioperative data.

Variable (*n* = 120)	Values
Volume of supplemental local anesthetic (mL)	8.2 (5–11.5 [3–13])
Number of patients requiring supplemental local anesthetic	78 (65%)
Volume of supplemental fentanyl (mcg)	65 (50–75)
Number of patients requiring intraoperative fentanyl	15 (12.5%)
Volume of tramadol (mL)	5 (3–8)
Number of patients requiring tramadol postoperatively	9 (7.5%)
Block performance time (min)	7.8 (6–8.5 [5–10])
Duration of surgery (min)	38 (32–42 [27–55])
Hospital stay (days)	1.5 (1–2 [1–3])
Postoperative VAS scores	
Postoperative 1 h	1.5 (0–3 [0–7])
Postoperative 6 h	1 (0–1.5 [0–4])
Postoperative 12 h	0.5 (0–1 [0–3])
Postoperative 24 h	0 (0–0 [0–0])

A comparison between patients receiving PECS II block who required intraoperative supplemental local anesthetic (*n* = 24) and those who did not (*n* = 96) did not show statistically significant differences in various factors, including age, gender, hypertension, coronary artery disease, diabetes mellitus, chronic renal failure, pacemaker type, ICD type, block performance time, duration of surgery, and hospital stay (Table 3).

**TABLE 3:** Comparison of variables between patients receiving PECS II block who required intraoperative supplemental local anesthetic and those who did not.

Variable	Supplementation (*n* = 24)	No Supplementation (*n* = 96)	*p*
Age (years)	66.5 ± 14.2	64.8 ± 15.1	0.652
Gender, male	16 (66.7%)	64 (66.7%)	1.000
Hypertension	12 (50%)	48 (50%)	1.000
Coronary artery disease	9 (37.5%)	36 (37.5%)	1.000
Diabetes mellitus	6 (25%)	24 (25%)	1.000
Chronic renal failure	1 (4.2%)	4 (4.2%)	1.000
Pacemaker	8 (33.3%)	32 (33.3%)	1.000
ICD	10 (41.7%)	40 (41.7%)	1.000
Block performance time (min)	7.0 ± 1.4	7.2 ± 1.6	0.754
Duration of surgery (min)	35.5 ± 6.1	36.1 ± 6.3	0.831
Hospital stay (days)	1.3 ± 0.5	1.2 ± 0.4	0.536

In Table 4, we present the results of both patient and implanting physician satisfaction levels with regard to Pain Management. Among the 120 patients surveyed, 36% reported being “Very Satisfied” with pain management, while 28% expressed “Satisfaction.” A smaller proportion of patients, 16%, reported feeling “Neutral,” and 9% were “Dissatisfied,” while an additional 11% were “Very Dissatisfied.” In contrast, among the 15 implanting physicians, 43% reported being “Very Satisfied” with pain management, 32% were “Satisfied,” and 11% felt “Neutral.” Both “Dissatisfaction” and “Very Dissatisfaction” were relatively low at 9% and 5%, respectively.

**TABLE 4:** Patients and physician's perception on pain management.

Satisfaction level	Patient satisfaction (%, *n* = 120)	Implanting physician satisfaction (%, *n* = 15)
Very satisfied	36	43
Satisfied	28	32
Neutral	16	11
Dissatisfied	9	9
Very dissatisfied	11	5

## 5 Discussion

In this study, we aimed to assess the effectiveness and feasibility of using the PECS II block during CIED insertion procedures. Our findings indicated that while the PECS II block alone may not provide complete surgical anesthesia, it did create suitable intraoperative conditions for CIED insertion, especially when supplemented with local anesthetic. Importantly, patients reported high levels of satisfaction with this approach, and it delivered effective postoperative analgesia for up to 24 h.

The PECS II block, a modification of the PECS I block, involves a two‐part injection process. The first part, equivalent to the PECS I block, targets the plane between the pectoralis major and minor muscles, where the medial and lateral pectoral nerves reside (Blanco, Fajardo, and Parras 2012). This provides analgesia to the anterior chest wall. In the second part, local anesthetic is injected into a deeper fascial plane between the pectoralis minor and serratus anterior muscles. This blocks the intercostobrachial nerve, lateral branches of the intercostal nerves, and the long thoracic nerve, extending analgesia to the anterolateral chest wall and adjacent axilla (Blanco, Fajardo, and Parras 2012).

While conventional transvenous CIED procedures may be manageable with PECS I block alone, our study suggests that the innervation of the chest wall is intricate, and PECS I block alone may not suffice for complete analgesia. Combinations of regional anesthesia techniques, such as the PECS I and transversus thoracic muscle plane block, have been explored for improved analgesia. However, accessing the transverse thoracic muscle by ultrasound can be challenging due to its thin structure and proximity to vital structures like blood vessels, pleura, and pericardium.

Our study demonstrates that the PECS II block alone can be a viable option for CIED insertion, offering broader anesthesia coverage compared to the PECS I block. While not previously well‐documented, a few case reports have shown promising results (Mavarez, Ripat, and Suarez 2019; Pai, Shariat, and Bhatt 2019). However, our study revealed that additional local anesthetic was needed in a significant portion of patients, particularly during the initial incision and dissection stages. This may be attributed to the block's limited spread to the skin incision level, which is typically a few centimeters beneath the clavicle.

It's worth noting that the optimal volume and concentration of local anesthetic for PECS II block remain areas of exploration. While larger volumes are generally associated with greater spread and nerve involvement, there is a lack of dose‐comparison studies to determine the ideal parameters. Our findings, as well as previous research, emphasize the potential of PECS II block in improving perioperative analgesia during CIED insertion procedures (Murata, Hida, and Hara 2016).

In our study, the PECS II block demonstrated its effectiveness in providing postoperative analgesia for up to 24 h, with an impressively high level of patient satisfaction. Remarkably, only two patients out of the entire cohort reported experiencing pain within the first 24 h after the procedure. These outcomes align closely with previous research, reinforcing the idea that the PECS II block can significantly reduce postoperative pain scores and substantially lower the overall need for opioid medication following CIED placement (Renzini et al. 2020).

Earlier investigations have also illustrated the value of PECS II block in postoperative pain management for CIED insertion. Yang et al. (2020) found that PECS II block reduced cumulative pain and mean opioid consumption during the initial 24 h after CIED placement, even in pediatric patients. Similarly, Elhaddad et al. (2023) reported that PECS II block not only lowered pain scores and opioid use but also contributed to stable hemodynamics in children undergoing subpectoral pacemaker implantation.

While CIED insertion is generally considered a minor procedure that can be conducted under local anesthesia, our study underscores the importance of postoperative pain management in this context. Patients' experiences of pain following CIED implantation can significantly impact their satisfaction, prolong hospital stays, and result in emotional distress (Wilson et al. 2021). It's worth noting that some centers may still face challenges in optimizing postoperative pain management for CIED procedures. Studies have indicated that opioid prescription rates could surpass 20% following CIED insertion, despite the relatively minor nature of the intervention (Lee et al. 2019).

These findings collectively highlight the need for the development of standardized guidelines for postoperative pain management in CIED insertion. Such guidelines would help eliminate variations in practice across different healthcare centers and ensure that patients consistently receive effective pain relief and optimal care in the postoperative phase.

This study is not without its limitations, and it is important to acknowledge these aspects to provide a comprehensive assessment of the research. Firstly, it is worth noting that we did not conduct a power analysis for this study, primarily because it was designed as a pilot study. This decision might have implications for the ability to detect smaller but potentially clinically significant differences. Another noteworthy limitation is the absence of a comparative group, which would have provided a more robust basis for validating the reported data. Without a control group, it becomes challenging to draw definitive conclusions regarding the specific impact of the PECS block on the observed outcomes. Additionally, the lack of blinding in this study presents a potential source of bias. Patients who were aware of receiving the PECS block might have reported experiencing less pain due to confirmation bias. Blinding, or a placebo control, could help mitigate this bias in future investigations. Furthermore, while no complications related to the PECS block were observed in our study, it is crucial to recognize that our sample size was relatively small. Consequently, it is not advisable to generalize the absence of complications to a larger population. Larger studies would be needed to assess the safety of the procedure more comprehensively. Despite these limitations, the data collected in this study can serve as a valuable foundation for powering and informing future controlled, randomized studies. These future studies could further explore the efficacy, safety, and patient experiences associated with the PECS block in the context of CIED insertion, potentially providing more robust evidence for its use in clinical practice.

## 6 Conclusion

In summary, our study demonstrates that the PECS II block, when used in conjunction with additional local anesthetic, offers effective postoperative pain relief for a duration of at least 24 h following CIED insertion. While it may not entirely replace the need for surgical anesthesia during the procedure, the PECS block does contribute to a more comfortable intraoperative experience for patients. To establish the robustness of these findings and provide a more definitive assessment, further controlled randomized studies are warranted. These future investigations will help solidify the role of the PECS II block in enhancing pain management and patient satisfaction in the context of CIED insertion.

## Author Contributions

S.Z. and J.M. provided the conceptualization. R.K., M.A.A., R.Z., and M.A. provided the methodology. A.M. was involved in supervision. M.A.A., M.A., A.M., W.A., and R.Z. provided the first draft. J.M., R.K., and S.Z. provided the final draft. S.Z., J.M., M.A.A., R.Z., M.A., A.M., and W.A. was involved in literature review.

## Conflicts of Interest

The authors declare no conflicts of interest.

## Data Availability

The data that support the findings of this study are available on request from the corresponding author. The data are not publicly available due to privacy or ethical restrictions.

## References

[anec70005-bib-0001] Ardon, A. E. , J. E. George 3rd , K. Gupta , et al. 2022. “The Use of Pectoralis Blocks in Breast Surgery: A Practice Advisory and Narrative Review From the Society for Ambulatory Anesthesia (SAMBA).” Annals of Surgical Oncology 29, no. 8: 4777–4786. 10.1245/s10434-022-11724-9. 35428960

[anec70005-bib-0002] Blanco, R. , M. Fajardo , and Maldonado T. Parras . 2012. “Ultrasound Description of Pecs II (Modified Pecs I): A Novel Approach to Breast Surgery.” Revista Española de Anestesiología y Reanimación 59, no. 9: 470–475. 10.1016/j.redar.2012.07.003. 22939099

[anec70005-bib-0003] Chakraborty, A. , R. Khemka , T. Datta , and S. Mitra . 2016. “COMBIPECS, the Single‐Injection Technique of Pectoral Nerve Blocks 1 and 2: A Case Series.” Journal of Clinical Anesthesia 35: 365–368. 10.1016/j.jclinane.2016.07.040. 27871558

[anec70005-bib-0004] Elhaddad, A. M. , S. M. Hefnawy , M. A. El‐Aziz , M. M. Ebraheem , and A. K. Mohamed . 2023. “Pectoral Nerve Blocks for Transvenous Subpectoral Pacemaker Insertion in Children: Randomized Controlled Trial.” Korean Journal of Anesthesiology 76: 424–432. 10.4097/kja.22681. 36632640 PMC10562074

[anec70005-bib-0005] Haas, S. , H. P. Richter , and J. C. Kubitz . 2009. “Anesthesia During Cardiologic Procedures.” Current Opinion in Anaesthesiology 22, no. 4: 519–523. 10.1097/ACO.0b013e32832dbad6. 19506472

[anec70005-bib-0006] Harding, M. E. 2015. “Cardiac Implantable Electronic Device Implantation: Intraoperative, Acute, and Remote Complications.” AACN Advanced Critical Care 26, no. 4: 312–319. 10.1097/NCI.0000000000000112. 26484991

[anec70005-bib-0007] Hoerner, E. , O. Stundner , F. Naegele , et al. 2023. “The Impact of PECS II Blockade in Patients Undergoing Minimally Invasive Cardiac Surgery – A Prospective, Randomized, Controlled, and Triple‐Blinded Trial.” Trials 24, no. 1: 570. 10.1186/s13063-023-07530-7. 37667362 PMC10476350

[anec70005-bib-0008] Jin, Z. , R. Li , T. J. Gan , Y. He , and J. Lin . 2020. “Pectoral Nerve (PECs) Block for Postoperative Analgesia – A Systematic Review and Meta‐Analysis With Trial Sequential Analysis.” International Journal of Physiology, Pathophysiology and Pharmacology 12, no. 1: 40–50. 32211121 PMC7076325

[anec70005-bib-0009] Kilin, M. , A. S. Kavakli , A. Karaveli , et al. 2023. “PECS II Block for Cardiac Implantable Electronic Device Insertion: A Pilot Study.” Pacing and Clinical Electrophysiology 46, no. 10: 1251–1257. 10.1111/pace.14811. 37665000

[anec70005-bib-0010] Lee, J. Z. , A. K. Pasha , A. E. Glasgow , et al. 2019. “Postoperative Opioid Prescription Patterns and New Opioid Refills Following Cardiac Implantable Electronic Device Procedures.” Heart Rhythm 16, no. 12: 1841–1848. 10.1016/j.hrthm.2019.08.011. 31648998

[anec70005-bib-0011] Malik, J. , N. Javed , G. Rana , M. Shoaib , U. Ishaq , and H. Chauhan . 2021. “Outcomes of Intracutaneous Sutures in Comparison With Intracutaneous Staples in Cardiac Implantable‐Electronic Device Pocket Closure.” Anatolian Journal of Cardiology 25, no. 10: 716–720. 10.5152/AnatolJCardiol.2021.96644. 34622786 PMC8504665

[anec70005-bib-0012] Mavarez, A. C. , C. I. Ripat , and M. R. Suarez . 2019. “Pectoralis Plane Block for Pacemaker Insertion: A Successful Primary Anesthetic.” Frontiers in Surgery 6: 64. 10.3389/fsurg.2019.00064. 31824958 PMC6879420

[anec70005-bib-0013] Moon, E. J. , S. B. Kim , J. Y. Chung , J. Y. Song , and J. W. Yi . 2017. “Pectoral Nerve Block (Pecs Block) With Sedation for Breast Conserving Surgery Without General Anesthesia.” Annals of Surgical Treatment and Research 93, no. 3: 166–169. 10.4174/astr.2017.93.3.166. 28932733 PMC5597541

[anec70005-bib-0014] Murata, H. , K. Hida , and T. Hara . 2016. “Transverse Thoracic Muscle Plane Block: Tricks and Tips to Accomplish the Block.” Regional Anesthesia and Pain Medicine 41, no. 3: 411–412. 10.1097/AAP.0000000000000374. 27093277

[anec70005-bib-0015] Pai, B. H. P. , A. N. Shariat , and H. V. Bhatt . 2019. “PECS Block for an ICD Implantation in the Super Obese Patient.” Journal of Clinical Anesthesia 57: 110–111. 10.1016/j.jclinane.2019.04.003. 30965271

[anec70005-bib-0016] Renzini, M. , U. Ripani , L. Golia , F. Nisi , and F. Gori . 2020. “Pectoralis (PecS) Nerve Block 1 for Port‐a‐Cath Removal and Central Venous Catheter (CVC) Replacement.” Medicinski Glasnik 17, no. 2: 352–355. 10.17392/1158-20. 32253905

[anec70005-bib-0017] Turkmen, S. , and M. Mutlu . 2022. “Evaluation of the Effect of Different Block Techniques on Open‐Heart Surgery in the Postoperative Period: A Prospective Observational Study.” Cardiovascular Journal of Africa 33, no. 3: 153–156. 10.5830/CVJA-2022-016. 35333279 PMC9540322

[anec70005-bib-0018] Wilson, D. G. , N. Brewster , R. J. Taylor , et al. 2021. “Pain During Cardiac Implantable Electronic Device Implantation.” British Journal of Cardiology 28, no. 4: 43. 10.5837/bjc.2021.043. 35747068 PMC9063704

[anec70005-bib-0019] Yalamuri, S. , R. Y. Klinger , W. M. Bullock , D. D. Glower , B. A. Bottiger , and J. C. Gadsden . 2017. “Pectoral Fascial (PECS) I and II Blocks as Rescue Analgesia in a Patient Undergoing Minimally Invasive Cardiac Surgery.” Regional Anesthesia and Pain Medicine 42, no. 6: 764–766. 10.1097/AAP.0000000000000661. 29016551

[anec70005-bib-0020] Yang, J. K. , D. S. Char , K. S. Motonaga , et al. 2020. “Pectoral Nerve Blocks Decrease Postoperative Pain and Opioid Use After Pacemaker or Implantable Cardioverter‐Defibrillator Placement in Children.” Heart Rhythm 17, no. 8: 1346–1353. 10.1016/j.hrthm.2020.03.009. 32201270

